# Associations between new-onset postoperative atrial fibrillation and long-term outcome in patients undergoing surgical aortic valve replacement

**DOI:** 10.1093/ejcts/ezad103

**Published:** 2023-03-24

**Authors:** Mary Rezk, Amar Taha, Susanne J Nielsen, Andreas Martinsson, Lennart Bergfeldt, Tomas Gudbjartsson, Stefan Franzén, Anders Jeppsson

**Affiliations:** Department of Molecular and Clinical Medicine, Institute of Medicine, Sahlgrenska Academy, University of Gothenburg, Gothenburg, Sweden; Department of Cardiothoracic Surgery, Region Västra Götaland, Sahlgrenska University Hospital, Gothenburg, Sweden; Department of Molecular and Clinical Medicine, Institute of Medicine, Sahlgrenska Academy, University of Gothenburg, Gothenburg, Sweden; Department of Cardiology, Region Västra Götaland, Sahlgrenska University Hospital, Gothenburg, Sweden; Department of Molecular and Clinical Medicine, Institute of Medicine, Sahlgrenska Academy, University of Gothenburg, Gothenburg, Sweden; Department of Cardiothoracic Surgery, Region Västra Götaland, Sahlgrenska University Hospital, Gothenburg, Sweden; Department of Molecular and Clinical Medicine, Institute of Medicine, Sahlgrenska Academy, University of Gothenburg, Gothenburg, Sweden; Department of Cardiology, Region Västra Götaland, Sahlgrenska University Hospital, Gothenburg, Sweden; Department of Molecular and Clinical Medicine, Institute of Medicine, Sahlgrenska Academy, University of Gothenburg, Gothenburg, Sweden; Department of Cardiology, Region Västra Götaland, Sahlgrenska University Hospital, Gothenburg, Sweden; Department of Cardiothoracic Surgery, Landspitali University Hospital, Reykjavik, Iceland; Faculty of Medicine, University of Iceland, Reykjavik, Iceland; Centre for Registries, Region Västra Götaland, Gothenburg, Sweden; Department of Molecular and Clinical Medicine, Institute of Medicine, Sahlgrenska Academy, University of Gothenburg, Gothenburg, Sweden; Department of Cardiothoracic Surgery, Region Västra Götaland, Sahlgrenska University Hospital, Gothenburg, Sweden

**Keywords:** Postoperative atrial fibrillation, Surgical aortic valve replacement, Coronary artery bypass grafting

## Abstract

**OBJECTIVES:**

Data on prognostic implications of new-onset postoperative atrial fibrillation (POAF) after surgical aortic valve replacement (SAVR) is limited. We sought to explore associations between POAF, early initiated oral anticoagulation (OAC) and long-term outcome after SAVR and combined SAVR + coronary artery bypass grafting (CABG).

**METHODS:**

This is a retrospective, population-based study including all isolated SAVR (*n* = 7038) and combined SAVR and CABG patients (*n* = 3854) without a history of preoperative atrial fibrillation (AF) in Sweden 2007–2017. Individual patient data were merged from 4 nationwide registries. Inverse probability of treatment weighting-adjusted Cox regression models were employed separately in SAVR and SAVR + CABG patients. The median follow-up time was 4.7 years (range 0–10 years).

**RESULTS:**

POAF occurred in 44.5% and 50.7% of SAVR and SAVR + CABG patients, respectively. In SAVR patients, POAF was associated with increased long-term risk of death [adjusted hazard ratio (aHR) 1.21 (95% confidence interval 1.06–1.37)], ischaemic stroke [aHR 1.32 (1.08–1.59)], any thromboembolism, heart failure hospitalization and recurrent AF. In SAVR + CABG, POAF was associated with death [aHR 1.31 (1.14–1.51)], recurrent AF and heart failure, but not with ischaemic stroke [aHR 1.04 (0.84–1.29)] or thromboembolism. OAC was dispensed within 30 days after discharge to 67.0% and 65.9%, respectively, of SAVR and SAVR + CABG patients with POAF. Early initiated OAC was not associated with reduced risk of death, ischaemic stroke or thromboembolism in any group of patients.

**CONCLUSIONS:**

POAF after SAVR is associated with an increased risk of long-term mortality and morbidity. Further studies are warranted to clarify the role of OAC in SAVR patients with POAF.

**Figure:**
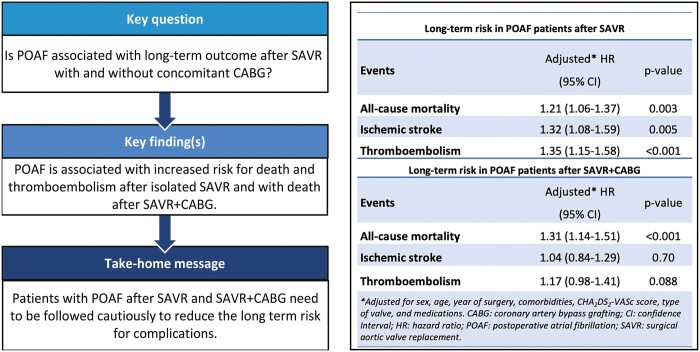

## INTRODUCTION

New-onset postoperative atrial fibrillation (POAF) is the most prevalent arrythmia after cardiac surgery, with a reported incidence of up to 50% after surgical aortic valve replacement (SAVR) [1–4]. In cardiac surgery patients, POAF has been associated with prolonged hospital stay, readmissions and short- and long-term morbidity and mortality [1–4]. However, most studies have been performed in coronary artery bypass grafting (CABG) patients while there are limited data on long-term outcome after POAF in patients undergoing SAVR.

Current European Society of Cardiology and European Association for Cardio-thoracic Surgery joint guidelines state that oral anticoagulation (OAC) therapy may be considered in patients with POAF after cardiac surgery (class IIb, level of evidence B), after taking stroke risk and bleeding risk into consideration [5]. This recommendation is based on a few retrospective studies involving cardiac surgery patients, while the remaining evidence come from non-surgical atrial fibrillation (AF) patients. No specific recommendations for OAC in SAVR patients with POAF have been published.

The objectives of the present study were to examine associations between POAF after SAVR and combined SAVR and CABG and the long-term risk of death, thromboembolic events, heart failure and recurrent AF, in a large nationwide study cohort. Furthermore, we investigated if early initiated OAC was associated with long-term risk of complications after SAVR.

## PATIENTS AND METHODS

### Ethics statement

This nationwide, observational, registry-based cohort study followed the 1975 Declaration of Helsinki and was approved by the Regional Human Research Ethics Committee in Gothenburg, Sweden (registration number 139-16, approved 4 April 2016). The committee waived the need for individual patient consent.

### Reporting

We have followed the recommendations in the Strengthening the Reporting of Observational Studies in Epidemiology statement in writing this manuscript [6].

### Study design and patient population

All Swedish residents 18 years old and older who underwent first-time isolated SAVR or SAVR and concomitant CABG from 1 January 2007 to 31 December 2017 were eligible. The patients were identified in the Swedish Cardiac Surgery Registry. Exclusion criteria were (i) previous history of AF, (ii) endocarditis during the last 6 months prior to surgery or (iii) death before postoperative day 31. A flowchart of included and excluded patients is depicted in Supplementary Material, Fig. S1.

POAF was defined as any new-onset POAF episode in a patient without documentation of AF before index hospitalization for the cardiac surgery. Patients were considered to have developed POAF if (i) POAF was reported in the SWEDEHEART registry during the index hospitalization, or (ii) had been given a new AF diagnosis in the Swedish National Patient Registry within the first 30 postoperative days, or (iii) had undergone electrical cardioversion during the index hospitalization as stated in the SWEDEHEART registry or Swedish National Patient Registry. Patients were followed until occurrence of death, emigration or the end of the study period (31 December 2017).

### Data sources

Individual patient information was retrieved from 4 mandatory registries and databases. The Swedish Cardiac Surgery Registry [7] is a part of the SWEDEHEART registry [8]. The registry contains detailed clinical and procedural information on all patients undergoing cardiac surgery in Sweden since 1992. Information about preoperative AF was obtained from the SWEDEHEART registry and the National Patient Registry, which was established in 1987 and covers all diagnoses including length of stay, and discharge dates in Sweden from all hospitalizations and hospital outpatient visits [9]. The diagnoses in the National Patient Registry have a validity of 85–95% [10]. All baseline information and outcome variables are reported according to the International Classification of Diseases, Tenth Revision. A list of the International Classification of Diseases, Tenth Revision codes used is given in Supplementary Material, Table S1.

The Dispensed Drug Registry was used to retrieve information on medications dispensed within 120 days before enrolment, as well as after discharge by using anatomical therapeutic chemical classification codes. A list of the anatomical therapeutic chemical codes used is presented in Supplementary Material, Table S2. Early initiated OAC therapy was defined as a dispensed prescription for OAC within 30 days after discharge. The National Cause of Death Registry provided information about mortality data. All registries utilized were linked into 1 database using the unique personal identification number all inhabitants in Sweden receive shortly upon birth or immigration.

### Outcome variables

The outcome variables reported are all-cause mortality, ischaemic stroke, thromboembolism (which was defined as either ischaemic stroke, transient ischaemic attack, or peripheral arterial embolization), heart failure, recurrent AF and major bleeding. An end point event was accounted for when reported in the National Patient Registry for the first time and met all the following criteria: (i) occurred after postoperative day 30, (ii) was associated with hospitalization and (iii) was reported as the primary or secondary diagnosis for the hospitalization. Recurrent AF was accounted for when associated with hospitalization in the National Patient Registry and registered as the primary diagnosis.

### Statistics

Continuous variables are presented as means and standard deviations and were compared using Student’s *t*-tests. Categorical variables were compared using chi-square tests and presented as numbers and percentages. The incidence rates were estimated as the number of events per 1000 person-years with 95% exact Poisson confidence intervals. POAF and non-POAF patients were compared before and after inverse probability of treatment weighting (IPTW), where the weights were based on propensity scores [11]. Supplementary Material, Table S3 shows the variables used in the propensity score matching. The variables include patient characteristics (e.g. age and sex), medical history, comorbidities, medications (including platelet inhibitors), CHA_2_DS_2_-VASc score, indication for surgery (aortic stenosis or regurgitation), year of surgery and type of prosthesis implanted (mechanical or biological). IPTW-adjusted Cox proportional hazards regression models were used to study the association between POAF and outcome variables. A generalized boosted regression model was used to estimate the propensity scores. The balance between the groups was determined by the average standardized mean difference across all cofounders included in the models. An absolute standardized difference ≤0.10 was considered sufficient. The absolute standardized difference before and after weighting in the study population is presented in Supplementary Material, Fig. S1. All statistical analyses were performed using R version 4.2.1 (R Foundation for Statistical Computing, Vienna, Austria). A *P*-value of <0.05 was considered statistically significant in all hypothesis testing, without any adjustment for multiple comparison.

## RESULTS

### Patients

A total of 14 038 patients underwent SAVR or SAVR + CABG in Sweden during the study period. After exclusion of patients who had preoperative AF (*n* = 2106), patients with endocarditis (*n* = 685) and patients who died within 30 days after surgery (*n* = 355), a total of 7038 SAVR patients and 3854 SAVR + CABG patients remained (Supplementary Material, Fig. S2). The median follow-up was 4.7 years (range 0–10 years).

### Isolated surgical aortic valve replacement

#### General

POAF was reported in 3131/7038 (44.5%) isolated SAVR patients. The baseline characteristics for SAVR patients with and without POAF are presented in Table 1. During follow-up, a total of 1055/7038 (15.0%) of the isolated SAVR patients died, 461 (6.6%) had an ischaemic stroke, 303 (4.3%) had a TIA, 708 (10.1%) suffered any thromboembolic event, 26 (0.4%) had a peripheral arterial embolism episode, 632 (9.0%) required hospitalization for heart failure and 2061 (29.3%) had at least 1 episode of AF (either recurrent in POAF patients or new-onset in non-POAF patients). Major bleeding occurred in 809 patients (11.5%).

**Table 1:** Preoperative characteristics in isolated surgical aortic valve replacement patients with and without postoperative atrial fibrillation

Variable	POAF, *n* = 3131 (44.5%)	No POAF, *n* = 3907 (55.5%)	*P*-Value
Female sex	1323 (42.3)	1658 (42.4)	0.90
Age (years)	72 ± 10	67 ± 13	<0.001
Body mass index (kg m^−2^)	27.5 ± 5.5	27.3 ± 5.9	0.16
Previous myocardial infarction	220 (7.0)	246 (6.3)	0.24
Diabetes mellitus	556 (17.8)	627 (16.0)	0.061
Hypertension	2021 (64.5)	2234 (57.2)	<0.001
Chronic respiratory disease	409 (13.1)	434 (11.1)	0.013
Peripheral vascular disease	223 (7.1)	225 (5.8)	0.023
History of cancer	621 (19.8)	555 (14.2)	<0.001
Renal failure	178 (5.7)	132 (3.4)	<0.001
Heart failure	575 (18.4)	590 (15.1)	<0.001
LVEF			0.093
>50%	2462 (78.6)	3134 (80.2)	
31–50%	507 (16.2)	605 (15.5)	
<30%	131 (4.2)	129 (3.3)	
Previous ischaemic stroke	244 (7.8)	229 (5.9)	0.002
Previous hemorrhagic stroke	22 (0.7)	16 (0.4)	0.13
Previous peripheral arterial embolism	13 (0.4)	15 (0.4)	0.99
CHA_2_DS_2_VASc			<0.001
≥2	2630 (84.0)	2830 (72.4)	
≥4	1389 (44.4)	1261 (32.3)	
Implantation of mechanical prosthesis	434 (13.9)	1025 (26.2)	<0.001
Implantation of biological prosthesis	2698 (86.2)	2884 (73.8)	<0.001
Indication for SAVR			
Stenosis	2696 (86.1)	3270 (83.7)	0.006
Regurgitation	335 (10.7)	490 (12.5)	0.019
Combined	185 (9.1)	376 (9.6)	0.48

The values are represented as means and standard deviations, or numbers and percentages.

LVEF: left ventricle ejection fraction; POAF: postoperative atrial fibrillation; SAVR: surgical aortic valve replacement.

#### Associations between postoperative atrial fibrillation and long-term outcome in surgical aortic valve replacement patients

**Figure 1:**
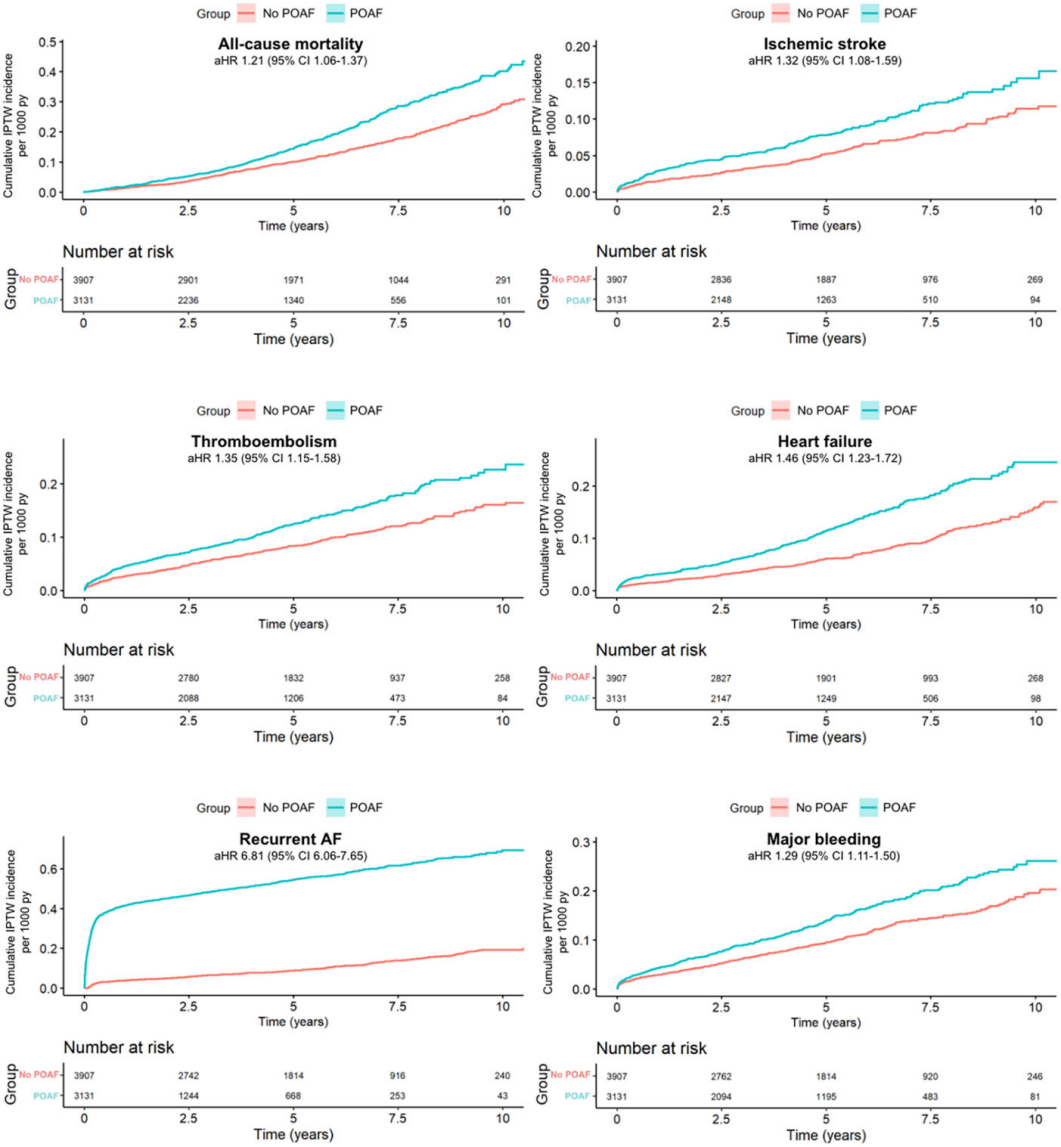
The adjusted cumulative incidence per 1000 patient years for all-cause mortality, ischaemic stroke, thromboembolism, heart failure hospitalization, recurrent atrial fibrillation and major bleeding in 3131 surgical aortic valve replacement patients with postoperative atrial fibrillation and 3907 patients with no postoperative atrial fibrillation.

Figure 1 shows the adjusted cumulative incidences of all-cause mortality, ischaemic stroke, thromboembolism, heart failure hospitalization, recurrent AF and major bleeding in patients who underwent isolated SAVR. Table 2 shows the incidence rate, unadjusted hazard ratios and adjusted hazard ratios. After adjustment, POAF in isolated SAVR patients was associated with a higher long-term risk of all-cause mortality, ischaemic stroke, thromboembolic event, heart failure hospitalization, recurrent AF and major bleeding.

**Table 2:** Incidence rates per 1000 patient years and unadjusted and adjusted associations between postoperative atrial fibrillation and long-term outcome after isolated surgical aortic valve replacement

Events	POAF incidence rate (95% Poisson CI)	No POAF incidence rate (95% Poisson CI)	Unadjusted HR (95% CI)	*P*-Value	Adjusted^a^ HR (95% CI)	*P*-Value
All-cause mortality	37.5 (34.4–40.8)	26.0 (23.8–28.3)	1.55 (1.38–1.75)	<0.001	1.21 (1.06–1.37)	0.003
Ischaemic stroke	17.5 (15.3–19.8)	11.5 (10.0–13.1)	1.53 (1.27–1.83)	<0.001	1.32 (1.08–1.59)	0.005
Thromboembolism	27.7 (24.9–30.7)	18.1 (16.2–20.1)	1.52 (1.31–1.76)	<0.001	1.35 (1.15–1.58)	<0.001
Heart failure	25.9 (23.2–28.7)	14.4 (12.8–16.2)	1.84 (1.57–2.16)	<0.001	1.46 (1.23–1.72)	<0.001
Recurrent atrial fibrillation	211.0 (201.0–221.4)	21.2 (19.2–23.4)	7.98 (7.15–8.91)	<0.001	6.81 (6.06–7.65)	<0.001
Major bleeding	30.9 (28.0–34.0)	21.3 (19.3–23.5)	1.45 (1.26–1.66)	<0.001	1.29 (1.11–1.50)	<0.001

Incidence rate per 1000 person-years with exact 95% Poisson confidence interval, unadjusted and IPTW-adjusted HRs with 95% CI. No POAF is used as reference. ^a^Adjusted for sex, age, year of surgery, comorbidities, CHA_2_DS_2_-VASc score, type of valve and medications. Supplementary Material, Table S3 shows a detailed list of variables used in the adjusted analysis.

CI: confidence interval; HR: hazard ratio; POAF: postoperative atrial fibrillation.

#### Anticoagulation

**Figure 2:**
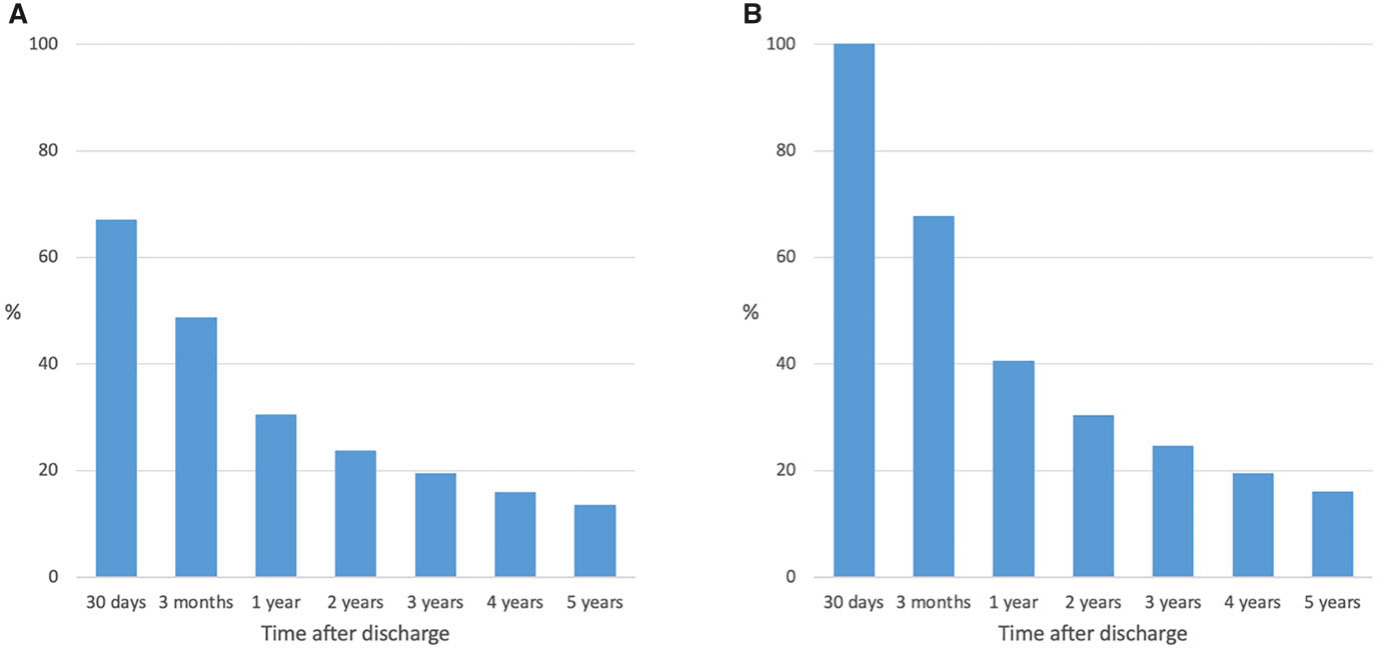
The proportion of postoperative atrial fibrillation patients dispensed with oral anticoagulation after isolated surgical aortic valve replacement at different time points (**A**) and the proportion of postoperative atrial fibrillation patients after isolated surgical aortic valve replacement with early initiated oral anticoagulation still treated at different time points (**B**).

Among all 3131 SAVR patients with POAF, a total of 2097 (67.0%) were dispensed OAC within 30 days after discharge. Baseline characteristics for POAF patients with and without early initiated OAC treatment are presented in Supplementary Material, Table S4. After 1 year, 30.5% of all POAF patients were dispensed OAC (Fig. 2A). Among POAF patients with early initiated OAC, 40.6% were still dispensed OAC after 1 year (Fig. 2B). The incidence rates and unadjusted and adjusted HR for complications in SAVR patients with early initiated OAC are shown in Supplementary Material, Table S5. After multivariable adjustment, early initiated OAC was not associated with reduced long-term risk for all-cause mortality, ischaemic stroke, thromboembolism or higher risk for major bleeding.

Platelet inhibitor(s) were dispensed to 2097/7038 (29.8%) of all the patients in the isolated SAVR group within 30 days after discharge, Supplementary Material, Table S6. At this time point, 870/3131 (27.8%) of the POAF patients in the isolated SAVR group were dispensed platelet inhibitor(s) and 241/3131 (7.7%) both OAC and platelet inhibitor(s), Supplementary Material, Table S7.

### Combined surgical aortic valve replacement and coronary artery bypass grafting

#### General

POAF was reported in 1954/3854 SAVR + CABG patients (50.7%). The baseline characteristics for patients with and without POAF after SAVR + CABG are presented in Table 3. During follow-up, a total of 910/3854 SAVR + CABG patients (23.6%) died, 384 (10.0%) had an ischaemic stroke, 195 (5.1%) had a TIA, 532 (13.8%) suffered any thromboembolic event, 569 (1.5%) required hospitalization for heart failure and 1243 (32.3%) had at least 1 episode of AF. Peripheral arterial embolism occurred in 28 (0.7%) patients, and major bleeding in 621 (16.1%).

**Table 3.** Preoperative characteristics in surgical aortic valve replacement + coronary artery bypass grafting patients with and without postoperative atrial fibrillation

Variable	POAF, *n* = 1954 (50.7%)	No POAF, *n* = 1900 (49.3%)	*P*-Value
Female sex	512 (26.2)	516 (27.2)	0.53
Age (years)	75 ± 7	73 ± 9	<0.001
Body mass index (kg m^−2^)	27.7 ± 9.8	27.7 ± 9.3	0.87
Previous myocardial infarction	496 (25.4)	473 (24.9)	0.75
Diabetes mellitus	542 (27.7)	542 (28.5)	0.61
Hypertension	1481 (75.8)	1349 (71.0)	0.001
Chronic respiratory disease	254 (13.0)	238 (12.5)	0.70
Peripheral vascular disease	217 (11.1)	179 (9.4)	0.095
History of cancer	381 (19.5)	359 (18.9)	0.66
Renal failure	187 (9.6)	101 (5.3)	<0.001
Heart failure	456 (23.3)	382 (20.1)	0.017
LVEF			0.82
>50%	1382 (70.7)	1347 (70.9)	
31–50%	445 (22.8)	435 (22.9)	
<30%	111 (5.7)	99 (5.2)	
Previous ischaemic stroke	218 (11.2)	178 (9.4)	0.076
Previous hemorrhagic stroke	10 (0.5)	14 (0.7)	0.50
Previous peripheral arterial embolism	12 (0.6)	6 (0.3)	0.26
CHA_2_DS_2_VASc			<0.001
≥2	1918 (98.2)	1834 (96.5)	
≥4	1441 (73.8)	1214 (63.9)	
Implantation of mechanical prosthesis	129 (6.6)	264 (13.9)	<0.001
Implantation of biological prosthesis	1825 (93.4)	1636 (86.1)	<0.001
Indication for SAVR			
Stenosis	1773 (90.7)	1684 (88.6)	0.036
Regurgitation	145 (7.4)	151 (7.9)	0.58
Combined	202 (10.3)	207 (10.9)	0.61

The values are represented as means and standard deviations, or numbers and percentages.

LVEF: left ventricle ejection fraction; POAF: postoperative atrial fibrillation; SAVR: surgical aortic valve replacement.

#### Associations between postoperative atrial fibrillation and long-term outcome in surgical aortic valve replacement *+* coronary artery bypass grafting patients

**Figure 3:**
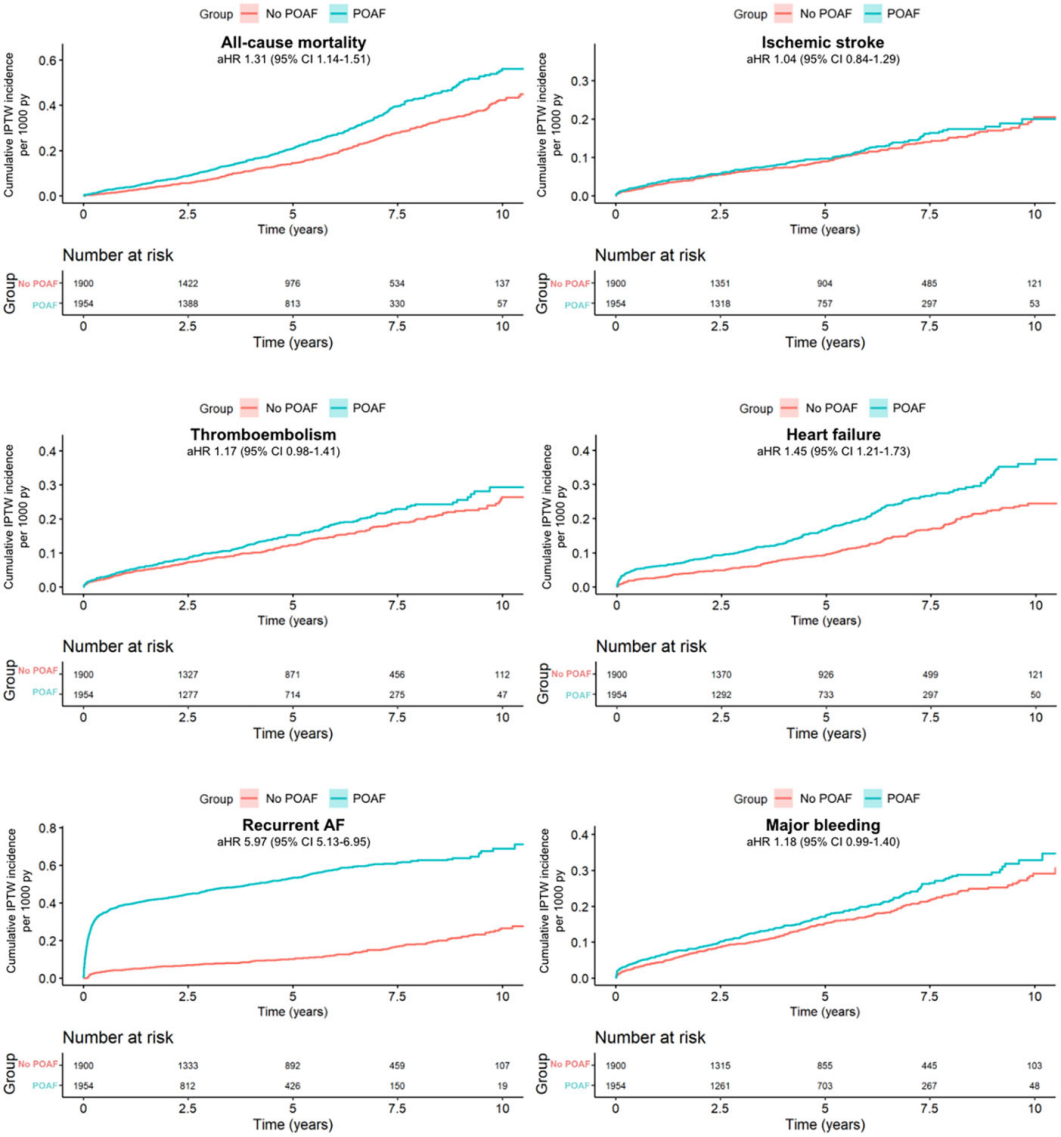
The adjusted cumulative incidence per 1000 patient years for all-cause mortality, thromboembolism, heart failure hospitalization and recurrent atrial fibrillation in 1954 coronary artery bypass grafting + surgical aortic valve replacement patients with postoperative atrial fibrillation and 1900 patients with no postoperative atrial fibrillation.

Figure 3 shows the adjusted cumulative incidences of all-cause mortality, ischaemic stroke, thromboembolism, heart failure hospitalization, recurrent AF and major bleeding in patients that underwent SAVR + CABG. Table 4 shows the incidence rates, unadjusted hazard ratios and adjusted hazard ratios. After adjustment, POAF after SAVR + CABG was associated with a higher risk of all-cause mortality, heart failure hospitalization and recurrent AF, but not with increased long-term risk of ischaemic stroke, thromboembolism or major bleeding.

**Table 4:** Incidence rates per 1000 patient years and unadjusted and adjusted associations between postoperative atrial fibrillation and long-term outcome after surgical aortic valve replacement + coronary artery bypass grafting

Events	POAF incidence rate (95% Poisson CI)	No POAF incidence rate (95% Poisson CI)	Unadjusted HR (95% CI)	*P*-Value	Adjusted^a^ HR (95% CI)	*P*-Value
All-cause mortality	57.4 (52.5–62.7)	41.6 (37.6–45.8)	1.51 (1.32–1.72)	<0.001	1.31 (1.14–1.51)	<0.001
Ischaemic stroke	22.9 (19.7–26.4)	20.9 (18.1–24.1)	1.10 (0.90–1.34)	0.38	1.04 (0.84–1.29)	0.70
Thromboembolism	34.5 (30.6–38.9)	28.3 (25.0–32.0)	1.22 (1.03–1.45)	0.024	1.17 (0.98–1.41)	0.088
Heart failure	41.5 (37.2–46.2)	24.8 (21.7–28.2)	1.72 (1.45–2.03)	<0.001	1.45 (1.21–1.73)	<0.001
Recurrent atrial fibrillation	199.1 (186.9–211.8)	26.3 (23.1–29.9)	6.25 (5.42–7.20)	<0.001	5.97 (5.13–6.95)	<0.001
Major bleeding	40.8 (36.5–45.5)	33.4 (29.8–37.4)	1.21 (1.04–1.42)	0.017	1.18 (0.99–1.40)	0.059

Incidence rate per 1000 person-years with exact 95% Poisson confidence interval, unadjusted and IPTW-adjusted HRs with 95% CI. No POAF is used as reference. ^a^Adjusted for sex, age, year of surgery, comorbidities, CHA_2_DS_2_-VASc score, type of valve and medications. Supplementary Material, Table S3 shows a detailed list of variables used in the adjusted analysis.

CI: confidence interval; HR: hazard ratio; IPTW: inverse probability of treatment weighting; POAF: Postoperative atrial fibrillation.

#### Anticoagulation

**Figure 4.**
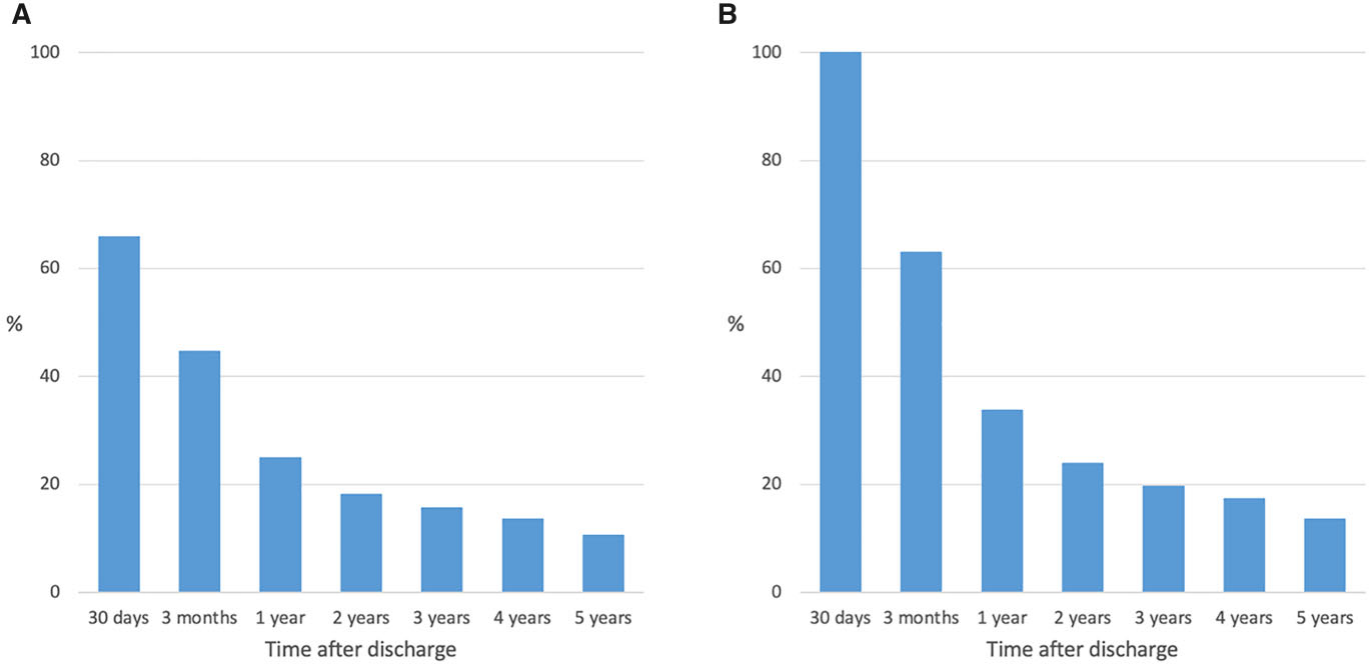
The proportion of postoperative atrial fibrillation patients dispensed with oral anticoagulation after surgical aortic valve replacement + coronary artery bypass grafting at different time points (**A**) and the proportion of postoperative atrial fibrillation patients after surgical aortic valve replacement + coronary artery bypass grafting with early initiated oral anticoagulation still treated at different time points (**B**).

Among all 1954 POAF patient who underwent CABG + SAVR, a total of 1287 (65.9%) received OAC treatment within 30 days after discharge. Baseline characteristics for POAF patients with and without early initiated OAC treatment are presented in Supplementary Material, Table S8. After 1 year, 24.9% of all POAF patients were dispensed OAC (Fig. 4A). Among POAF patients with early initiated OAC, 33.8% were still dispensed OAC after 1 year (Fig. 4B). The incidence rate, unadjusted HR and adjusted HR for long-term complications in SAVR + CABG patients with early initiated anticoagulation are shown in Supplementary Material, Table S9. No association between early OAC therapy and long-term risk for all-cause mortality, ischaemic stroke, thromboembolism or major bleeding was observed.

Platelet inhibitor(s) were dispensed to 1669/3854 (43.3%) of all the patients in the SAVR + CABG group within 30 days after discharge (Supplementary Material, Table S6). At this time point, 781/1954 (40.0%) of the POAF patients in the SAVR + CABG group were dispensed platelet inhibitor(s) and 412/1954 (21.1%) both OAC and platelet inhibitor(s) (Supplementary Material, Table S7).

## DISCUSSION

The main findings of this large retrospective population-based cohort study were that POAF after isolated SAVR was associated with increased long-term risk for all-cause mortality, ischaemic stroke, thromboembolic events, heart failure hospitalization, recurrent AF and major bleeding, while POAF after SAVR + CABG was associated with death, heart failure and recurrent AF.

The incidences of POAF in patients undergoing SAVR (44.5%) and SAVR + CABG (50.7%) in the present study are comparable to what previously have been reported. Saxena *et al.* [3] found an incidence of 35.1% in a multicentre study of isolated SAVR patients operated 2001–2009. Kalra *et al.* [1] reported an incidence of 50.1% in a very large US database which included 122 765 hospitalizations 2012–2015. In SAVR + CABG populations, reports are rare. Saxena *et al.* [12] reported a POAF incidence of 44.1% in a study of 2028 patients while studies in valve + CABG patients, sometimes without distinction about type of valve, have reported incidences similar to that in isolated SAVR [4, 13].

Only a few studies have studied the association between POAF and outcome in SAVR patients and long-term mortality. In the present study, we observed a significant association between POAF and an increased long-term risk for death in both isolated SAVR and SAVR + CABG patients. This result contrasts to previous studies in isolated SAVR populations by Saxena *et al.* [3] and Swinkels *et al.* [14], where no difference in adjusted long-term mortality between POAF and no POAF SAVR patients was observed. The difference may at least partly be explained by the size of the study cohorts. Our study included 7038 patients while the previous studies were markedly smaller [3, 14].

In SAVR + CABG patients, there were in contrast to isolated SAVR patients, no significant associations between POAF and thromboembolic complications, including ischaemic stroke. This may be explained by the more frequent use of platelet inhibitors in SAVR + CABG patients with POAF (Supplementary Material, Table S7). One year after surgery 59.0% of the POAF patients in the SAVR + CABG group received platelet inhibitors compared to 33.8% in isolated SAVR patients. In patients with non-surgical non-valvular AF, it has been demonstrated in a meta-analysis that platelet inhibition is associated with a 22% reduction in the risk for stroke [15]. However, corresponding data on patients with POAF after cardiac surgery are lacking.

The 2020 European Society of Cardiology/European Association for Cardio-thoracic Surgery guidelines [5] state that OAC therapy may be considered in patients with POAF after cardiac surgery if they are at risk for stroke. The recommendation is rather weak (class IIb) and based on limited evidence (level B). In SAVR patients, there is even less evidence. Butt *et al.* [4] found that OAC was associated with a reduced risk for thromboembolic events in patients undergoing left-sided valve surgery, but studies in pure SAVR and SAVR + CABG populations are lacking. In the present study, two-thirds of SAVR and SAVR + CABG patients were treated with early initiated OAC which is similar to the study by Butt *et al.* [4]. The high incidence in our study is partly explained by the fact that we included all SAVR patients in Sweden during the study period and this includes also patients who received a mechanical aortic valve prosthesis, where OAC is indispensable. Furthermore, some centres in Sweden are also treating the majority of patients receiving a biological aortic valve prosthesis with OAC for a limited period of time, most often 3 months [16]. It was not possible, on an individual level, to distinguish between OAC as a treatment for POAF or as a part of local protocol for bioprostheses. One may argue that patients that received OAC for other reasons than POAF should be excluded from further analyses, but except for mechanical prostheses, it is not possible to distinguish these patients in the registers. Instead, we chose a pragmatic strategy and included all SAVR and SAVR + CABG patients with POAF and compared those who received OAC with those who did not, independently of indication for OAC.

In the present study, we did not find that early initiated OAC was associated with a reduced risk for death or thromboembolic events, neither in isolated SAVR nor in SAVR + CABG patients. These findings should not be interpreted as an argument against OAC treatment in SAVR patients with POAF, given the observational study design and the limited treatment periods, as illustrated in Figs 2 and 4. There are subgroups of SAVR patients with POAF where OAC therapy most likely is essential for reducing the thromboembolic risk. The decision to treat POAF patients with OAC should be individualized, taking both the risk for thromboembolic and bleeding complications into considerations, as recommended in current guidelines [5]. Further studies, preferably prospective randomized trials, investigating the role of OACs in POAF patients after SAVR and SAVR + CABG are warranted.

An increased risk of heart failure hospitalization and AF recurrence in POAF patients, were observed in the present study. The increased risk for heart failure is in accordance with recent studies both in CABG patients [17, 18] and in mixed cardiac surgery and non-cardiac surgery patients [19], but has not previously been reported after SAVR. AF and heart failure are closely related conditions with partly the same risk factors and pathophysiology. It might be difficult to determine which condition precipitates or exacerbates the other. Furthermore, it has in fact been speculated that heart failure and AF are 2 expressions of the same underlying pathology [20]. The prevalence of AF in patients with heart failure exceeds 50% regardless of the ejection fraction [21].

### Limitations and strengths

The present study has a number of important limitations. The observational study design infers a risk for selection bias and residual confounding. We did not adjust for type and size of the implanted valves or centre. The limitations concerning OAC treatment are discussed above. Additionally, it was not possible to identify the exact time period between the development of POAF and the initiation of OAC treatment. The data on recurrent AF during follow-up are most likely underestimated due to the prevalence of asymptomatic AF. The strengths of this study include the large nationwide study population, the real-life setting, the use of IPTW matching and the use of validated registries. The size of the study population allowed us to study isolated SAVR and SAVR + CABG patients separately.

## CONCLUSIONS

New-onset POAF after isolated SAVR is associated with an increased long-term risk for death, ischaemic stroke, thromboembolic complications, heart failure, recurrent AF and major bleeding. After SAVR + CABG, POAF is associated with death, heart failure and recurrent AF. Patients with POAF after SAVR need to be followed cautiously to reduce the risk for complications. The role of OAC therapy in SAVR and SAVR + CABG patients with POAF but without indispensable indication for OAC remains unclear.

## Supplementary Material

ezad103_Supplementary_Data

## Data Availability

The data underlying this article were provided by SWEDEHEART and national healthcare registries in Sweden. Data will be shared on reasonable request to the corresponding author with permission of SWEDEHEART and the Swedish National Board of Health and Welfare.

## Notes

Presented at the EACTS Annual Meeting, Milan, Italy, 5-8 October 2022.

## ABBREVIATIONS

AF: Atrial fibrillation
aHR: Adjusted hazard ratio
CABG: Coronary artery bypass grafting
IPTW: Inverse probability of treatment weighting
OAC: Oral anticoagulation
POAF: Postoperative atrial fibrillation
SAVR: Surgical aortic valve replacement
