# Supplementary material for: Associations between new-onset postoperative atrial fibrillation and long-term outcome in patients undergoing surgical aortic valve replacement
Source: Eur J Cardiothorac Surg. 2023 Mar 24;63(5):ezad103. doi: 10.1093/ejcts/ezad103 (PMC10229100; doi:10.1093/ejcts/ezad103)
Supplement: ezad103_Supplementary_Data [file ezad103_supplementary_data.docx]

**SUPPLEMENTARY MATERIAL**

[Supplementary Table 1. Diagnoses according to ICD-10 codes 2](#_Toc123821470)

[Supplementary Table 2. Medications according to ATC-codes 3](#_Toc123821471)

[Supplementary Table 3. Variables included in the propensity score adjusted analyses 3](#_Toc123821472)

[Supplementary Table 4. Preoperative characteristics in SAVR patients with postoperative atrial fibrillation and with and without early initiated oral anticoagulation 5](#_Toc123821473)

[Supplementary Table 5. Incidence rates per 1000 patient years and unadjusted and adjusted associations between early initiated OAC and long-term outcome after isolated surgical aortic valve replacement 6](#_Toc123821474)

[Supplementary Table 6. The proportion of all patients dispensed with oral anticoagulation, antiplatelets and a combination of oral anticoagulation and antiplatelets after SAVR and SAVR+CABG over time 7](#_Toc123821475)

[Supplementary Table 7. The proportion of POAF patients dispensed with oral anticoagulation, antiplatelets and a combination of oral anticoagulation and antiplatelets after SAVR and SAVR+CABG over time 8](#_Toc123821476)

[Supplementary Table 8. Preoperative characteristics in SAVR+CABG patients with postoperative atrial fibrillation and with and without early initiated oral anticoagulation 9](#_Toc123821477)

[Supplementary Table 8. Incidence rates per 1000 patient years and unadjusted and adjusted associations between early initiated OAC and long-term outcome after CABG and/or surgical aortic valve replacement 10](#_Toc123821478)

[Supplementary Figure 1. A flowchart of included and excluded patients 11](#_Toc123821479)

[Supplementary Figure 2. Unweighted and weighted absolute standard difference 12](#_Toc123821480)

# **Supplementary Table 1.** Diagnoses according to ICD-10 codes.

| Diagnose | ICD-10 |
| --- | --- |
| Intracranial bleeding | I60, I61, I62, I690, I691, I692 |
| Gastrointestinal bleeding | I850, I983, K226, K250, K252, K254, K256, K260, K262, K264, K266, K270, K272, K274, K276, K280, K284, K290, K625, K661, K920, K921, K922, K25, K26, K27, K28, I850, I983, K221,K226 |
| Hemopericardium | I230, I312 |
| Hemothorax | J942 |
| Urogenital bleeding | N02, R319, N95, N939, N501A |
| Other bleeding | H431, R04, R58, D629, T810, DR029, D50 |
| Anemia | D50-64 |
| Atrial fibrillation | I48 |
| Ischemic stroke | I63, I69.3, I69.4 |
| Stroke unspecified | I64 |
| TIA | G45, I66, I65 |
| Peripheral arterial embolism | I74 |
| Heart failure | I50, I110, I130, I132, I255, I42-43 |
| Hypertension | I10-15 |
| Diabetes mellitus | E10-14 |
| Ischemic heart disease | I20, I24, I25 |
| Myocardial infarction | I21, I22 |
| PCI | FNG |
| Peripheral vascular disease | I70, I71, I72, I73, I74, I77 |
| Vascular disease | I21, I22, I252, I70-73 |
| Renal disease and RRT | N17-19, DR016, DR024, KAS10, KAS20 |
| Liver disease | K70-77, JJB, JJC |
| Alcohol | E244, F10, G312, G621, G721, I426, K292, K70, K860, O354, P043, Q860, T51, Y90-91, Z502, Z714 |
| COPD | J44 |
| Pulmonary embolism | I26 |
| DVT | I80 |
| *COPD: Chronic obstructive pulmonary disease; DVT: Deep vein thrombosis; ICD-10: International classification of diseases 10th version; PCI: Percutaneous coronary intervention; TIA: Transitory ischemic attack.* | |

# **Supplementary Table 2.** Medications according to ATC-codes.

| Medication | ATC-codes |
| --- | --- |
| Antiarrhythmic drugs | C01B, C07AA07 |
| Antidiabetics | A10 |
| Anti-inflammatory and antirheumatic agents | M01 |
| Antiplatelets | B01AC, N01bA |
| Betablockers | C09 (excluding C07AA07) |
| Calcium antagonists | C08 |
| Digoxin | C01AA05 |
| Diuretics | C03 (excluding C03DA) |
| Lipid lowering agents | C10 |
| Mineralcorticoid receptor antagonists | C03DA |
| Oral anticoagulants | B01AA, B01AE, B01AF |
| Renin-angiotensin inhibitors | C09 |
| Systemic corticoids | H01 |
| *ATC: The Anatomical Therapeutic Classification* | |

# **Supplementary Table 3.** Variables included in the propensity score adjusted analysis

| Sex |
| --- |
| Age |
| Year of cardiac surgery |
| Body mass index |
| History of smoking |
| History of myocardial infarction |
| History of Non ST elevation myocardial infarction |
| History of ST elevation myocardial infarction |
| History of unstable angina |
| History of stable angina |
| History of chronic ischemic heart disease |
| Previous Percutaneous coronary intervention |
| History of diabetes mellitus |
| History of hypertension |
| History of chronic respiratory disease |
| History of congestive heart failure |
| History of peripheral arterial disease |
| History of cancer |
| History of alcohol |
| History of chronic renal failure |
| History of renal replacement therapy |
| History of heart failure |
| History of cardiomyopathy |
| Left ventricular ejection fraction |
| Preoperative atrial fibrillation |
| History of unspecified stroke |
| History of ischemic stroke |
| History of hemorrhagic stroke |
| History of transitory ischemic attack |
| History of subarachnoid bleeding |
| History of liver disease |
| History of liver operation |
| History of pulmonary embolism |
| History of deep vein thrombosis |
| History of peripheral arterial embolism |
| History of intracranial bleeding |
| History of gastrointestinal bleeding |
| History of anemia |
| History of pericardial bleeding |
| History of pulmonary bleeding |
| CHA2DS2-VASc score |
| History of endocarditis |
| History of aortic endocarditis |
| History of rheumatic cardiomyopathy |
| History of rheumatic aortic stenosis |
| History of rheumatic mitral valve |
| History of rheumatic bicuspid valve |
| History of dementia (including vascular dementia, Parkinson dementia and Alzheimer) |
| History of delirium |
| History of depression |
| Treatment with diuretics |
| Treatment with Mineralocorticoid receptor antagonists |
| Treatment with Beta-blockers |
| Treatment with Angiotensin-converting enzyme inhibitors |
| Treatment with Angiotensin receptor blockers |
| Treatment with Calcium channel blockers |
| Treatment with Lipid lowering agents |
| Treatment with Antiplatelets |
| Treatment with Oral anticoagulants |
| Treatment with Corticosteroids |
| Treatment with Non-steroidal anti-inflammatory drugs |
| Treatment with Antiarrhythmic agents |
| Treatment with Insulin and Antidiabetic agents |
| Implantation of mechanic aortic prothesis |
| Implantation of biologic aortic prothesis |
| Aortic stenosis |
| Aortic insufficiency |
| A combination of Aortic stenosis and aortic insufficiency |
| Body surface area |

# **Supplementary Table 4.** Preoperative characteristics in SAVR patients with postoperative atrial fibrillation and with and without early initiated oral anticoagulation.

| Variable | OAC  (n=2097) | No OAC  (n=1034) | p-value |  |
| --- | --- | --- | --- | --- |
| Female sex | 867 (41.3) | 456 (44.1) | 0.15 |  |
| Age [years] | 71 ± 10 | 73± 9 | <0.001 |  |
| Body mass index [kg*m-2] | 28 ± 6 | 27± 5 | 0.27 |  |
| Myocardial infarct | 147 (7.0) | 73 (7.1) | 1.000 |  |
| Diabetes mellitus | 359 (17.1) | 197 (19.1) | 0.20 |  |
| Hypertension | 1343 (64.0) | 678 (65.6) | 0.42 |  |
| Chronic respiratory disease | 268 (12.8) | 141 (13.6) | 0.54 |  |
| Peripheral vascular disease | 147 (7.0) | 76 (7.4) | 0.78 |  |
| History of cancer | 405 (19.3) | 216 (20.9) | 0.32 |  |
| Renal failure | 96 (4.6) | 82 (7.9) | <0.001 |  |
| Heart failure | 404 (19.3) | 197 (19.1) | 0.93 |  |
| LVEF |  |  | 0.608 |  |
| >50% | 1648 (78.6) | 814 (78.7) |  |  |
| 31-50% | 333 (15.9) | 174 (16.8) |  |  |
| <30% | 174 (8.3) | 39 (3.8) |  |  |
| Previous ischemic stroke | 167 (8.0) | 77 (7.4) | 0.66 |  |
| Previous hemorrhagic stroke | 15 (0.7) | 7 (0.7) | 1.000 |  |
| Previous TIA | 148 (7.1) | 70 (6.8) | 0.82 |  |
| Previous peripheral atrial embolism | 8 (0.4) | 5 (0.5) | 0.90 |  |
| CHA_2_DS_2_VASc |  |  | 0.031 |  |
| ≥2 | 1729 (82.5) | 901 (87.1) |  |  |
| ≥4 | 895 (42.7) | 494 (47.8) |  |  |
| Implantation of mechanical prosthesis | 407 (19.4) | 0 | <0.001 |  |
| Implantation of biological prosthesis | 1690 (80.6) | 1034 (100) | <0.001 |  |
| Indication for SAVR  Stenosis  Regurgitation  Combined | 1  790 (85.4)  246 (11.7)  190 (9.1) | 906 (87.6)  89 (8.6)  95 (9.2) | 0.096  0.009  0.96 |  |
| *Means and standard deviations, or numbers and percentages.*  *OAC: oral anticoagulants; TIA: transient ischemic attack.* | | | | |

#

#

# **Supplementary Table 5.** Incidence rates per 1000 patient years and unadjusted and adjusted associations between early initiated OAC and long-term outcome after isolated surgical aortic valve replacement.

| Events | OAC incidence rate (95% Poisson CI) | No OAC incidence rate (95% Poisson CI) | Unadjusted HR (95% CI) | p-value | Adjusted HR (95% CI) | p-value |  |
| --- | --- | --- | --- | --- | --- | --- | --- |
| All-cause mortality | 36.5 (32.8-40.5) | 39.7 (34.0-46.0) | 0.85 (0.71-1.02) | 0.087 | 0.88 (0.67-1.16) | 0.378 |  |
| Ischemic stroke | 17.2 (14.6-20.1) | 18.0 (14.2-22.6) | 0.95 (0.72-1.25) | 0.703 | 0.99 (0.72-1.37) | 0.962 |  |
| Thromboembolism | 28.4 (25.0-32.1) | 26.2 (21.5-31.6) | 1.09 (0.87-1.36) | 0.476 | 1.00 (0.73-1.38) | 0.983 |  |
| Major bleeding | 31.1 (27.6-34.9) | 30.5 (25.4-36.3) | 1.02 (0.83-1.26) | 0.848 | 1.14 (0.87-1.48) | 0.344 |  |
| *Incidence rate per 1000 person-years with exact 95% Poisson confidence interval, unadjusted and IPTW adjusted Hazard ratios with 95% confidence interval. No OAC is used as reference. Hazard ratios adjusted for sex, age, year of surgery, comorbidities, CHA_2_DS_2_-VASc score, type of valve, and medications. Supplementary Table 3 shows a detailed list of variables used in the adjusted analysis.*  *CI: Confidence interval; HR: Hazard ratio; OAC: Oral anticoagulants.* | | | | | | | |

# **Supplementary Table 6.** The proportion of all patients dispensed with oral anticoagulation, antiplatelets and a combination of oral anticoagulation and antiplatelets after SAVR and SAVR+CABG over time.

| Variable | SAVR (n=7038) | SAVR+CABG (n=3854) |
| --- | --- | --- |
| Oral anticoagulation  30 days  3 months  1 year  2 years  3 years  4 years  5 years | 4282 (60.9)  3150 (44.8)  1897 (27.0)  1544 (22.0)  1294 (18.4)  1095 (15.6)  966 (13.7) | 2261 (58.7)  1527 (39.6)  765 (19.9)  595 (15.5)  518 (13.5)  452 (11.7)  380 (9.9) |
| Antiplatelets  30 days  3 months  1 year  2 years  3 years  4 years  5 years | 2097 (29.8)  1767 (25.1)  2502 (35.6)  2340 (33.3)  1908 (27.1)  1615 (23.0)  1335 (19.0) | 1669 (43.3)  1532 (39.8)  2215 (57.5)  1951 (50.6)  1627 (42.2)  1352 (35.1)  1062 (27.6) |
| Oral anticoagulation + antiplatelets  30 days  3 months  1 year  2 years  3 years  4 years  5 years | 580 (8.3)  417 (5.9)  130 (1.9)  79 (1.1)  71 (1.0)  69 (1.0)  46 (0.7) | 761 (19.8)  567 (14.7)  215 (5.6)  99 (2.6)  87 (2.3)  55 (1.4)  42 (1.1) |
| *Numbers and percentages.  CABG: Coronary artery bypass grafting; SAVR: surgical aortic valve replacement.* | | |

# **Supplementary Table 7.** The proportion of POAF patients dispensed with oral anticoagulation, antiplatelets and a combination of oral anticoagulation and antiplatelets after SAVR and SAVR+CABG over time.

| Variable | SAVR (n=3131) | SAVR+CABG (n=1954) |
| --- | --- | --- |
| Oral anticoagulation  30 days  3 months  1 year  2 years  3 years  4 years  5 years | 2097 (67.0)  1524 (48.7)  954 (30.5)  742 (23.7)  607 (19.4)  496 (15.9)  420 (13.4) | 1287 (65.9)  873 (44.7)  486 (24.9)  355 (18.2)  305 (15.6)  265 (13.6)  206 (10.6) |
| Antiplatelets  30 days  3 months  1 year  2 years  3 years  4 years  5 years | 870 (27.8)  708 (22.6)  1013 (32.4)  976 (31.2)  790 (25.2)  646 (20.6)  503 (16.1) | 781 (40.0)  668 (34.2)  1021 (52.3)  882 (45.1)  734 (37.6)  587 (30.1)  433 (22.2) |
| Oral anticoagulation + antiplatelets  30 days  3 months  1 year  2 years  3 years  4 years  5 years | 241 (7.7)  178 (5.7)  46 (1.5)  30 (1.0)  25 (0.8)  34 (1.1)  23 (0.7) | 412 (21.1)  328 (16.8)  131 (6.7)  50 (2.6)  41 (2.1)  20 (1.0)  20 (1.0) |
| *Numbers and percentages.  CABG: Coronary artery bypass grafting; SAVR: surgical aortic valve replacement.* | | |

# **Supplementary Table 8.** Preoperative characteristics in SAVR+CABG patients with postoperative atrial fibrillation and with and without early initiated oral anticoagulation.

| Variable | OAC  (n=1287) | No OAC  (n=667) | p-value |  |
| --- | --- | --- | --- | --- |
| Female sex | 318 (24.7) | 194 (29.1) | 0.042 |  |
| Age [years] | 75 ± 7 | 76 ± 7 | 0.025 |  |
| Body mass index [kg*m-2] | 285 ± 12 | 274 ± 5 | 0.14 |  |
| Myocardial infarct | 315 (24.5) | 181 (27.1) | 0.22 |  |
| Diabetes mellitus | 339 (26.3) | 203 (30.4) | 0.062 |  |
| Hypertension | 993 (77.2) | 488 (73.2) | 0.058 |  |
| Chronic respiratory disease | 161 (12.5) | 93 (13.9) | 0.41 |  |
| Peripheral vascular disease | 132 (10.3) | 85 (12.7) | 0.11 |  |
| History of cancer | 247 (19.2) | 134 (20.1) | 0.68 |  |
| Renal failure | 90 (7.0) | 97 (14.5) | <0.001 |  |
| Heart failure | 302 (23.5) | 154 (23.1) | 0.90 |  |
| LVEF |  |  | 0.829 |  |
| >50% | 911 (70.8) | 471 (70.6) |  |  |
| 31-50% | 293 (22.8) | 152 (22.8) |  |  |
| <30% | 70 (5.4) | 41 (6.2) |  |  |
| Previous ischemic stroke | 139 (10.8) | 79 (11.8) | 0.54 |  |
| Previous hemorrhagic stroke | 4 (0.3) | 6 (0.9) | 0.16 |  |
| Previous TIA | 113 (8.8) | 57 (8.5) | 0.93 |  |
| Previous peripheral atrial embolism | 8 (0.6) | 4 (0.6) | 1.00 |  |
| CHA_2_DS_2_VASc |  |  | 0.16 |  |
| ≥2 | 1259 (97.8) | 659 (98.8) |  |  |
| ≥4 | 937 (72.8) | 504 (75.6) |  |  |
| Implantation of mechanical prosthesis | 116 (9.0) | 0 | <0.001 |  |
| Implantation of biological prosthesis | 1171 (91.0) | 667 (100) | <0.001 |  |
| Indication for AVR  Stenosis  Regurgitation  Combined | 1174 (91.2)  100 (7.8)  122 (9.5) | 599 (89.8)  45 (6.7)  80 (12.0) | 0.35  0.47  0.098 |  |
|  |  |  |  |  |
| *Means and standard deviations, or numbers and percentages.*  *OAC: oral anticoagulants; TIA: transient ischemic attack.* | | | | |

# **Supplementary Table 9.** Incidence rates per 1000 patient years and unadjusted and adjusted associations between early initiated OAC and long-term outcome after CABG and/or surgical aortic valve replacement.

| Events | OAC incidence rate (95% Poisson CI) | No OAC incidence rate (95% Poisson CI) | Unadjusted HR (95% CI) | p-value | Adjusted HR (95% CI) | p-value |  |
| --- | --- | --- | --- | --- | --- | --- | --- |
| All-cause mortality | 56.4 (50.6-62.8) | 59.5 (50.7-69.4) | 0.87 (0.72-1.05) | 0.16 | 1.04 (0.83-1.31) | 0.71 |  |
| Ischemic stroke | 21.0 (17.4-25.1) | 27.2 (21.1-34.4) | 0.77 (0.57-1.04) | 0.089 | 0.82 (0.58-1.17) | 0.27 |  |
| Thromboembolism | 32.1 (27.5-37.2) | 40.1 (32.5-48.8) | 0.80 (0.63-1.03) | 0.081 | 0.78 (0.58-1.06) | 0.11 |  |
| Major bleeding | 40.5 (35.4-46.3) | 41.4 (33.7-50.3) | 0.98 (0.77-1.24) | 0.87 | 0.98 (0.75-1.28) | 0.87 |  |
| *Incidence rate per 1000 person-years with exact 95% Poisson confidence interval, unadjusted and IPTW adjusted Hazard ratios with 95% confidence interval. No OAC is used as reference. Hazard ratios adjusted for sex, age, year of surgery, comorbidities, CHA_2_DS_2_-VASc score, type of valve, and medications. Supplementary Table 3 shows a detailed list of variables used in the adjusted analysis.*  *CI: Confidence interval; HR: Hazard ratio; OAC: Oral anticoagulants.* | | | | | | | |

**
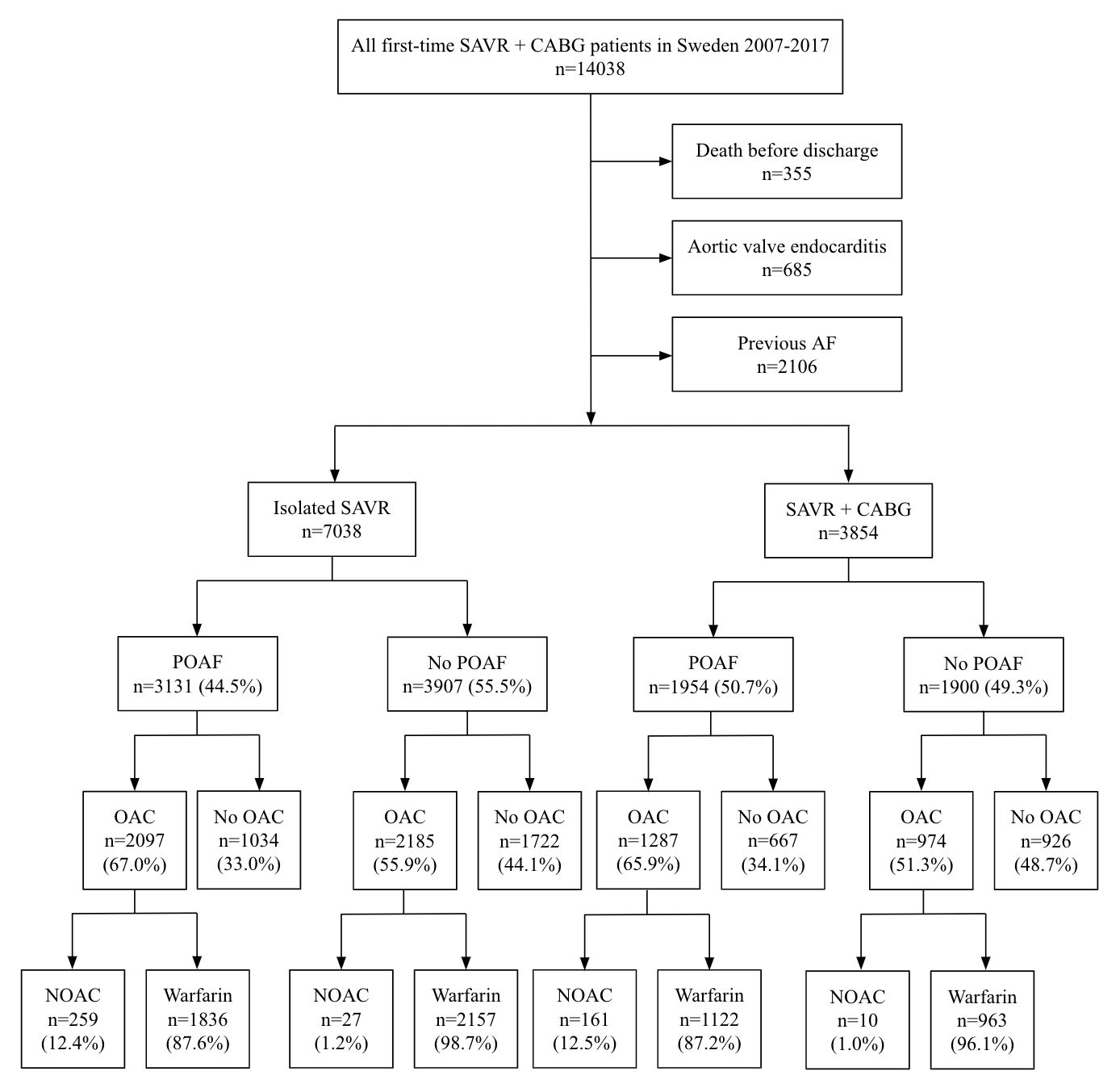
**

**Supplementary Figure** 1. A flowchart of included and excluded patients.
AF: Atrial fibrillation; CABG: Coronary artery bypass grafting; NOAC: Non-vitamin k antagonist oral anticoagulants: OAC: Oral anticoagulation; POAF: postoperative atrial fibrillation; SAVR: Surgical aortic valve replacement.


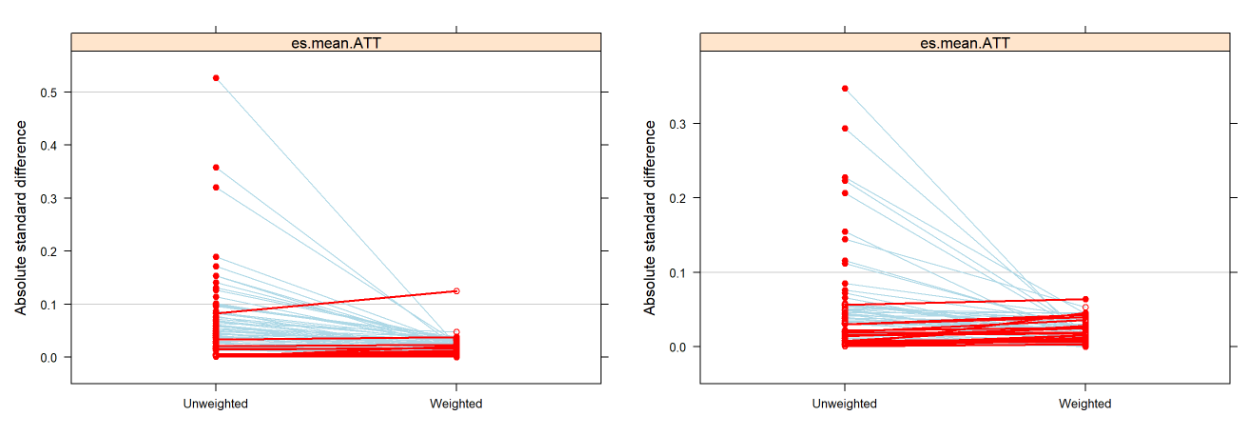


# **Supplementary Figure 2**. Unweighted and weighted absolute standard difference in isolated aortic valve replacement patients (left figure) and CABG and surgical aortic valve replacement patient (right figure).
